# The Capacity to Repair Sperm DNA Damage in Zygotes is Enhanced by Inhibiting WIP1 Activity

**DOI:** 10.3389/fcell.2022.841327

**Published:** 2022-04-05

**Authors:** Jiyeon Leem, Guang-Yu Bai, Jeong Su Oh

**Affiliations:** ^1^ Department of Integrative Biotechnology, Sungkyunkwan University, Suwon, South Korea; ^2^ Biomedical Institute for Convergence at SKKU (BICS), Sungkyunkwan University, Suwon, South Korea

**Keywords:** WIP1, sperm dna damage, DNA repair, zygote, zygotic genome activation

## Abstract

Maintaining genome integrity in germ cells is essential not only for successful fertilization and embryo development, but also to ensure proper transmission of genetic information across generations. However, unlike oocytes, sperm are incapable of repairing DNA damage. Therefore, sperm DNA damage is repaired after fertilization in zygotes using maternal DNA repair factors. In this study, we found that zygotic repair of paternal DNA damage is enhanced by inhibiting WIP1 activity. Oxidative stress induced DNA damage in sperm and severely impaired motility. Although DNA damage in sperm did not compromise fertilization, it increased DNA damage in the paternal pronucleus of zygotes. However, WIP1 inhibition during fertilization reduced DNA damage in the paternal pronucleus, improving the rate of two-cell development, and subsequent zygotic genome activation. Therefore, our results suggest that WIP1 inhibition could enhance maternal DNA repair capacity and thereby decrease paternal DNA damage in zygotes.

## Introduction

In order to preserve genomic integrity, eukaryotic cells have evolved a specialized network named DNA damage response (DDR). In response to DNA double-strand breaks (DSBs), ataxia telangiectasia-mutated (ATM) kinase phosphorylates the histone variant H2AX at serine 139 at the break sites, which in turn forms γ-H2AX foci. This event is essential to sustain the recruitment of downstream DDR factors in order to ensure repair of damaged DNA. After completion of DSB repair, activity of the DDR signaling is terminated by protein phosphatases to return cells to a pre-stress state, which is mainly achieved by wild-type p53-induced phosphatase 1 (WIP1) (Lu et al., 2008; Cha et al., 2010). WIP1 dephosphorylates numerous targets of the ATM-dependent signaling pathway, including p53, γ-H2AX, CHK1/2, and possibly also other proteins involved in DDR signaling (Yamaguchi et al., 2005). Moreover, alterations in WIP1 expression can abrogate the homeostatic balance, which is associated with tumorigenesis. Indeed, WIP1 was found to be frequently overexpressed in multiple cancers and was reported to act as oncogene. Conversely, WIP1 depletion delayed the onset of tumor development (Pechackova et al., 2017). Therefore, WIP1 has been known as a homeostatic regulator in DSB repair signaling.

Maintaining genomic integrity in germ cells is important not only for successful fertilization and embryo development, but also to ensure proper transmission of genetic information across generations. However, mature sperm are vulnerable to accumulating DNA damage because their capacity to repair DNA damage declines during spermatogenesis with the compaction of the chromatin by protamine and decrease in the cytoplasmic content, which limits access of repair factors to the damaged DNA (Ward and Coffey, 1991; Baarends et al., 2001). For this reason, DNA lesions are observed more frequently in mature sperm than in mature oocytes (Gonzalez-Marin et al., 2012). This DNA damage in sperm is considered as one of the major causes of male infertility and adversely impacts reproductive outcomes, including the transmission of paternal mutations (Evenson et al., 1980; Menezo et al., 2010).

Unlike sperm, mammalian oocytes are equipped with DNA repair machinery and capable of repairing DNA damage (Stringer et al., 2018). This maternal DNA repair machinery persists to sustain the zygote until its genome is fully activated, which occurs at the two-cell stage in mouse and later in human embryos (Braude et al., 1988; Hamatani et al., 2004). As mature sperm have reduced DNA repair capacity, it has been suggested that sperm DNA damage is repaired in the zygote using maternal DNA repair machinery (Brandriff and Pedersen, 1981). Indeed, mouse zygotes have been shown to recognize and repair DNA damage derived from sperm (Gawecka et al., 2013). Therefore, repair of sperm DNA damage seems to be a key factor to ensure a proper embryo development, especially when paternal DNA integrity is compromised.

We have previously reported that WIP1 inhibition enhances the DNA repair capacity of oocytes during prophase I arrest (Leem et al., 2018). Therefore, we investigated whether WIP1 inhibition could reduce the paternal DNA damage in zygotes after fertilization. In this study, we showed that WIP1 inhibition decreases DNA damage derived from sperm in zygotes by enhancing maternal DNA repair capacity of oocytes and zygotes.

## Materials and Methods

### Reagents

All chemicals and reagents were purchased from Sigma-Aldrich unless otherwise stated.

### Animals

All procedures for mouse care and use were conducted in accordance with the guidelines and approved by the Institutional Animal Care and Use Committees of Sungkyunkwan University (SKKUIACUC-2021-01-54-1). B6D2F1 (C57BL/6N x DBA/2) mice were purchased from Koatech Laboratory Animals, Inc. (Korea).

### Sperm Collection and Treatment

Sperm were collected from eight-week-old male mice from the cauda epididymis and allowed to swim out for 15 min in M2 medium (Sigma-Aldrich) supplemented with 0.4% bovine serum albumin (BSA) at 37°C as described previously (Fernandez-Gonzalez et al., 2019). To induce oxidative stress, sperm were spilt into culture medium containing different concentrations of hydrogen peroxide (H_2_O_2_; 0, 25, 50, or 100 μM) and cultured for 1 h at 37°C in 5% CO_2_ atmosphere.

### Oocyte Collection, ICSI, and Embryo Culture

Oocytes were isolated from six-week-old female mice super-ovulated by intraperitoneal injection of 5 IU pregnant mare serum gonadotrophin (PMSG), followed 48–52 h later by 5 IU human chorionic gonadotrophin (hCG). Ovulated oocytes at metaphase II (MII) stage were collected in M2 medium at 15 h post-hCG injection. After a brief exposure to hyaluronidase to remove cumulus cells, denuded MII oocytes were collected and used for subsequent intracytoplasmic sperm injection (ICSI) with a piezoelectric actuator as described previously (Bai et al., 2020). Briefly, the sperm head was isolated from the tail by applying one or more piezo pulses to the head-tail junction, and the sperm head was injected into an MII oocyte. After 30 min incubation at room temperature for recovery, sperm-injected oocytes were cultured in Continuous Single Culture Complete medium (Irvine Scientific) at 37°C in a humidified atmosphere containing 5% CO2. For WIP1 inhibition, sperm-injected oocytes were treated with 5 μM GSK2830371 (TOCRIS) dissolved in DMSO for 9 h in culture medium after ICSI. For control, an equal volume of DMSO was added to the media. The dose of DMSO used is standard in the field and is below 0.05% final concentration.

### Immunofluorescence Analysis

For immunofluorescence assays, sperm or embryos were fixed with 4% paraformaldehyde in phosphate buffered saline (PBS) for 10 min and permeabilized with 0.1% Triton X-100 and 0.01% Tween-20 at room temperature for 20 min. After blocking with 3% BSA-supplemented PBS for 1 h, samples were incubated with anti-γ-H2AX (1:250, Abcam), anti-8-OHdG (1:500, Abcam), or anti-Zscan4 (1:500, Abcam) antibodies overnight at 4°C. After extensively washing, samples were then incubated for 2 h with Alexa Fluor-conjugated 488 and 594 secondary antibodies (Jackson ImmunoResearch) at room temperature. Finally, samples were counterstained with DAPI and observed under a laser scanning confocal microscope (LSM700, Zeiss) equipped with a C-Apochromat 63 ×/1.2 water immersion objective. For measurement of immunofluorescence intensity, the mean intensity of fluorescence signals was measured after capturing at the same laser power. ZEN 2012 Blue (Zeiss) and ImageJ software (National Institutes of Health) were used for data analyses under the same processing parameters.

### Terminal Deoxynucleotidyl Transferase dUTP Nick End Labeling (TUNEL) Assay

The TUNEL assay was performed using an *In Situ* Cell Death Detection kit (Roche) according to the manufacturer’s instruction. Briefly, sperm suspension was smeared on slides and fixed for 10 min by ice-cold acetone-methanol (1:1) wet fixation and dried, as described previously (Antalikova et al., 2020). Acetone-fixed sperm were then washed three times with PBS and permeabilized on ice with 0.15% Triton-X100 and 0.1% sodium citrate for 1 h. Sperm were incubated with fluorescent-conjugated terminal deoxynucleotide transferase dUTP for 2 h at 37°C. After counterstaining with DAPI, sperm were mounted on glass slides, and the fluorescence intensity was measured using a LSM 700 laser scanning confocal microscope (Zeiss).

### Statistical Analysis

Statistical analysis was performed with GraphPad Prism Version 5.0 (GraphPad Software). Data are presented as means +SEM from at least three independent experiments, and each experimental group contained at least 15 embryos. Student’s t-test and one-way ANOVA with Tukey’s post-hoc test were used for differences between two groups and comparisons between more than two groups, respectively. A *p*-value less than 0.05 (*p* < 0.05) was considered statistically significant.

## Results and Discussion

### Oxidative Stress Induces DNA Damage in Sperm, Impairing Sperm Motility

**FIGURE 1:**
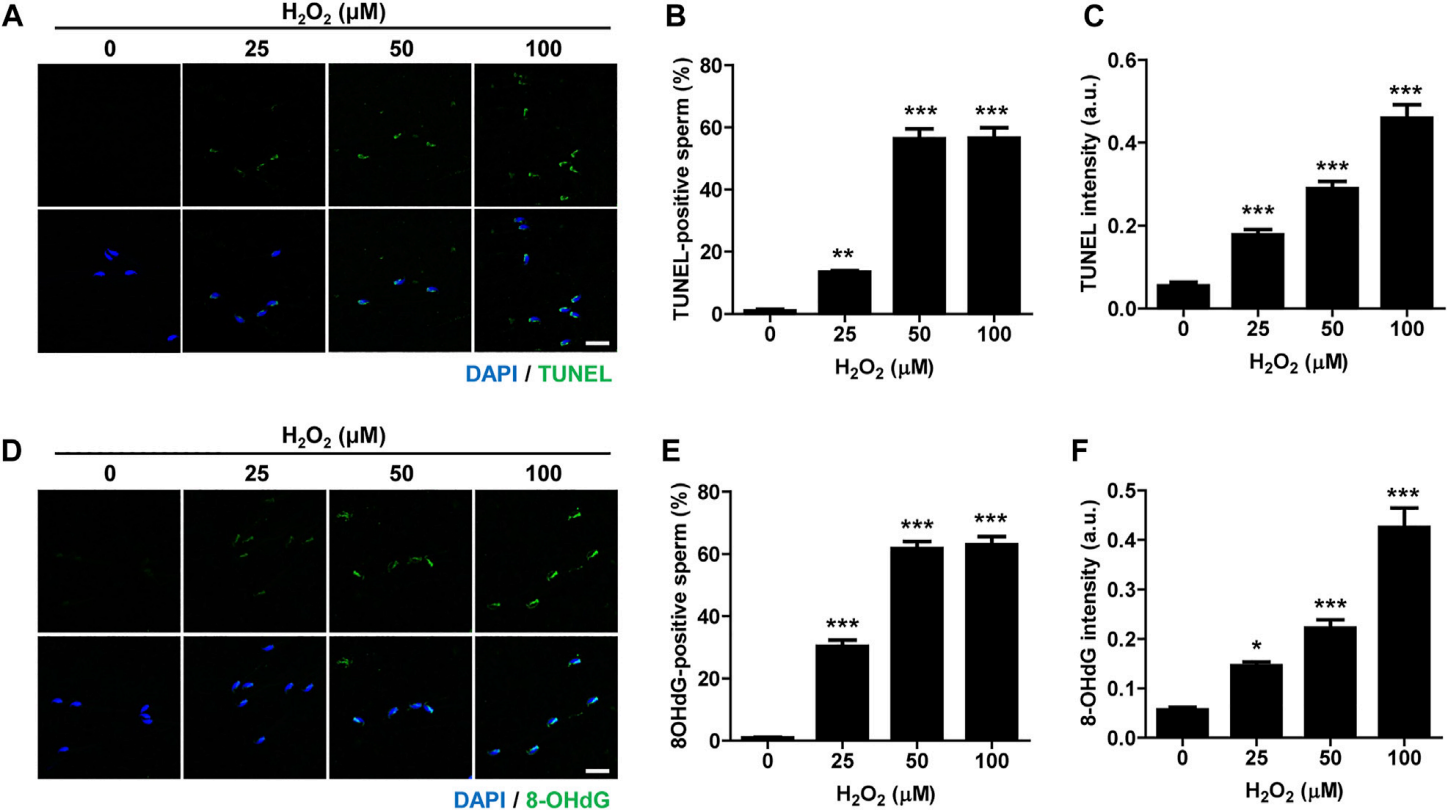
Oxidative stress induces DNA damage in sperm and impairs sperm motility. Sperm were treated with 0, 25, 50, and 100 μM hydrogen peroxide (H_2_O_2_) for 1 h. Sperm DNA fragmentation was assessed by TUNEL assay and 8-OHdG staining. **(A–C)** TUNEL-positive sperm and TUNEL intensity were quantified and shown with representative images. **(D–F)** 8-OHdG-positive sperm and 8-OHdG fluorescence intensity were quantified and shown in representative images. Scale bar, 10 μm. Data are expressed as mean +SEM. *p < 0.05, **p < 0.001, and ***p < 0.0001, compared to control (0).

Oxidative stress is considered the major cause of DNA damage in spermatozoa (Aitken and De Iuliis, 2010). Therefore, we treated mature, cauda-isolated sperm with different concentrations of hydrogen peroxide (H_2_O_2_), and determined the level of DNA damage. The number of TUNEL-positive sperm was elevated by increasing the concentration of H_2_O_2_ (Figures 1A,B). Similarly, the intensity of TUNEL signal increased proportionally with increase in H_2_O_2_ concentration (Figures 1A,C). To further confirm the sperm DNA damage induced by H_2_O_2_, we measured the level of 8-hydroxy-2′-deoxyguanosine (8-OHdG), a useful biomarker of oxidative DNA damage to sperm, which is formed by oxidative attack and has been highly correlated with sperm DNA fragmentation (De Iuliis et al., 2009). Consistent with the increase in TUNEL signal, the number of 8-OHdG-positive sperm and overall intensity of 8-OHdG signals increased proportionally to increase of H_2_O_2_ concentration (Figures 1D–F). Moreover, H_2_O_2_ treatment dramatically decreased sperm motility, increasing the number of non-motile sperm and impairing sperm aggregation (Supplementary Figure S1; Supplementary Movies S1, S2). Therefore, our data suggest that oxidative stress induces DNA damage in sperm and adversely affects sperm motility.

### WIP1 Inhibition Decreases Paternal DNA Damage in Zygotes

**FIGURE 2:**
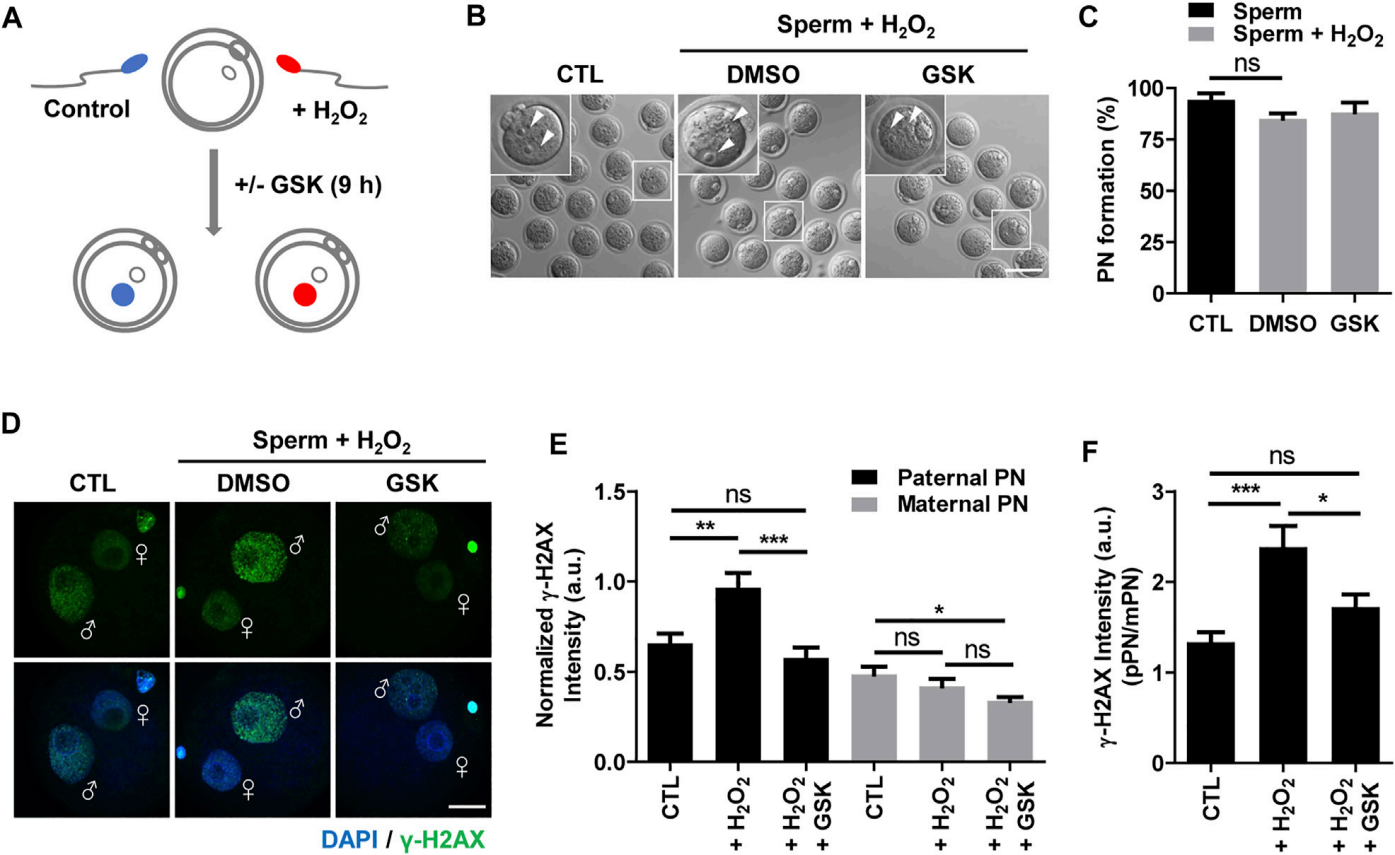
WIP1 inhibition enhances DNA damage repair in zygotes. **(A)** Schematic diagram of the experiment. After insemination with sperm treated with 100 μM hydrogen peroxide (H_2_O_2_) for 1 h, oocytes were cultured with or without GSK for 9 h. Fresh sperm were used as a control. **(B,C)** The rate of pronuclear (PN) formation was shown with representative images at 9 h after ICSI. Scale bar, 100 μm. Arrowheads indicate pronucleus. **(D,E)** The γ-H2AX intensity in paternal and maternal pronuclei was normalized to the mean DAPI intensity of two independent experiments and shown with representative images. ♂ and ♀ indicate the paternal PN and maternal PN, respectively. Scale bar, 20 μm. **(F)** The γ-H2AX intensity ratio of the paternal/maternal pronuclei was quantified. Data are expressed as mean +SEM. *p < 0.05, **p < 0.001, and ***p < 0.0001, ns; not significant.

To investigate the impact of WIP1 inhibition on DNA damage in sperm after fertilization, we performed ICSI with sperm after treating with H_2_O_2_. For WIP1 inhibition, we added a specific inhibitor, GSK2830371, in the culture medium of oocytes after the ICSI procedure for 9 h (Figure 2A) (Gilmartin et al., 2014; Leem et al., 2018). Control oocytes were treated with DMSO. Initial studies with just DMSO suggested no difference compared to unexposed oocytes (data not shown). Interestingly, we did not observe remarkable changes in the percentage of pronuclei formation (Figures 2B,C), which is consistent with previous report that oxidative damage to DNA in sperm did not preclude pronuclei formation at ICSI (Twigg et al., 1998). We next measured γ-H2AX signals in the paternal and maternal pronuclei in zygotes derived from sperm exposed to H_2_O_2_. As expected, the γ-H2AX intensity significantly increased in the paternal pronucleus but not in the maternal pronucleus. Notably, however, WIP1 inhibition during fertilization significantly decreased the γ-H2AX intensity in the paternal pronucleus (Figures 2D–F). Interestingly, the γ-H2AX intensity in the maternal pronucleus was slightly but significantly reduced by WIP1 inhibition (Figures 2D–F), implying that WIP1 inhibition improves the repair capacity of zygotes.

### WIP1 Inhibition Restores ZGA Impaired by Oxidative Stress on Sperm

**FIGURE 3:**
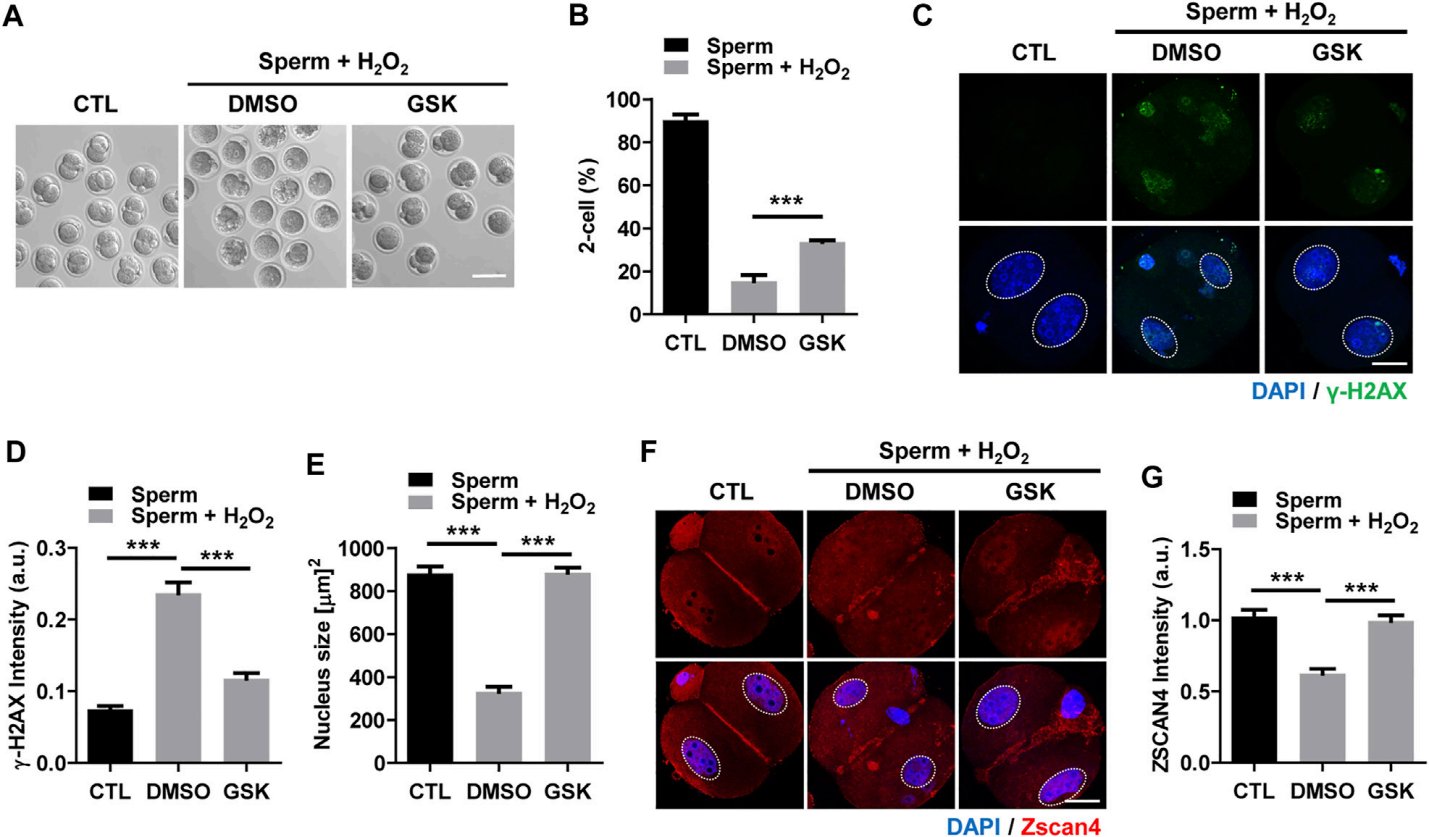
WIP1 inhibition improves two-cell development and zygotic genome activation. **(A,B)** The rate of two-cell embryos was shown with representative images at 24 h after ICSI. Scale bar, 100 μm. **(C–E)** The γ-H2AX intensity and nuclear size were quantified and shown with representative images. Scale bar, 20 μm. **(F,G)** The Zscan4 intensity was quantified and shown with representative images. Scale bar, 20 μm.

**FIGURE 4:**
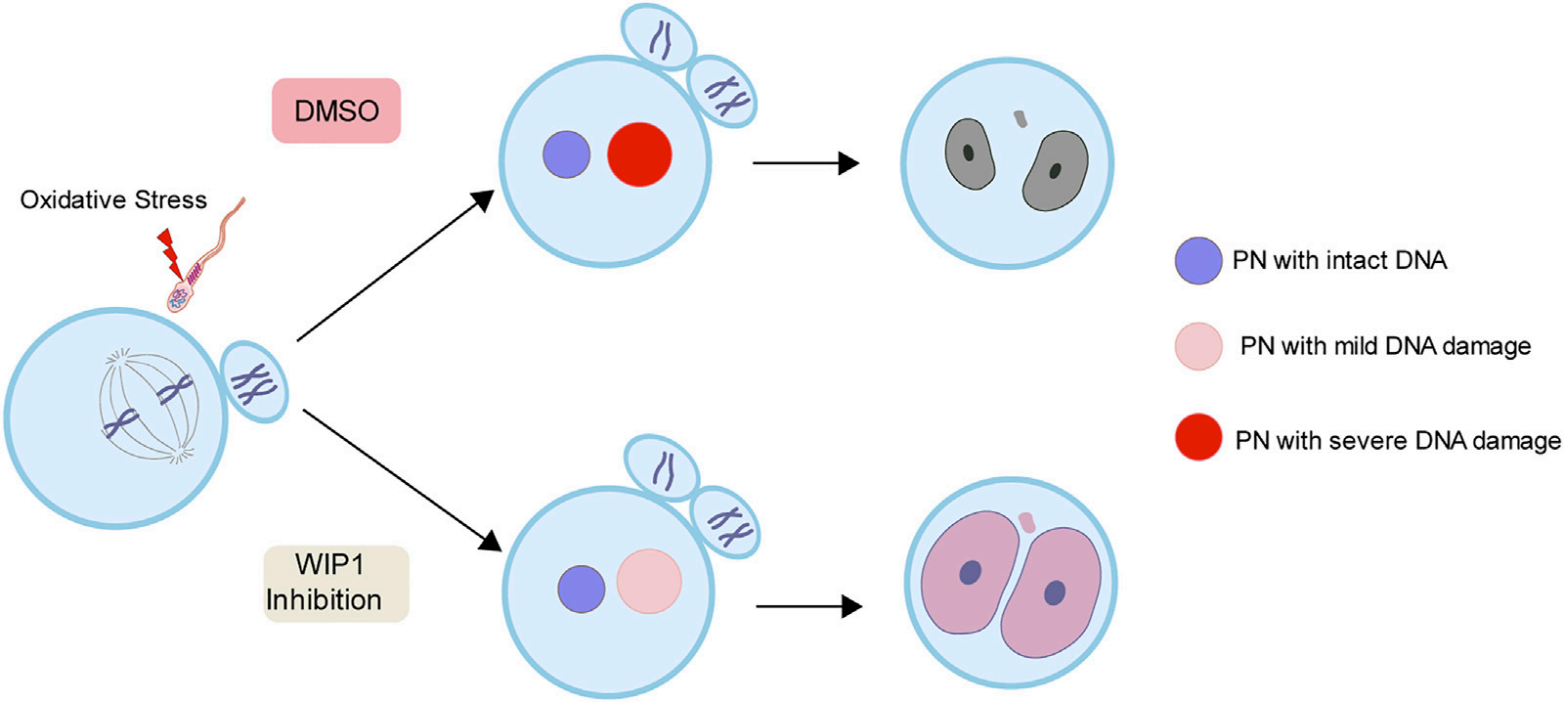
A working model for the effect of WIP1 inhibition on the repair of sperm-derived DNA damage in zygotes. Oxidative stress in sperm increases paternal DNA damage in zygotes and causes ZGA failure. However, WIP1 inhibition during fertilization reduces paternal DNA damage in zygotes and restores ZGA. PN, pronucleus.

To further investigate the impact of the decrease in DNA damage in the paternal pronucleus induced by WIP1 inhibition on embryonic development, we evaluated the *in vitro* development of zygotes. While zygotes containing the paternal DNA damage developed barely to the two-cell stage, the percentage of two-cell embryos increased after WIP1 inhibition (Figures 3A,B). Moreover, the γ-H2AX intensity decreased significantly in two-cell embryos by inhibiting WIP1 (Figures 3C,D). Interestingly, the size of the nucleus of two-cell embryos derived from sperm exposed to H_2_O_2_ decreased. However, the shrinkage of nuclear size was rescued by WIP1 inhibition (Figures 3C,E). Because zygotic genome activation (ZGA) occurs during the two-cell stage in the mouse (Hamatani et al., 2004), we asked whether the decreased nuclear size is associated with impaired ZGA. Therefore, we examined the expression of Zscan4 (zinc finger and scan domain-containing protein 4), which is expressed specifically during ZGA in the late two-cell stage of mouse embryos (Falco et al., 2007; Ko, 2016). Indeed, Zscan4 was expressed in the nucleus of two-cell embryos, but expression was barely detectable in embryos derived from sperm exposed to H_2_O_2_. Notably, this impaired expression of Zscan4 was rescued by WIP1 inhibition (Figures 3F,G), suggesting that WIP1 inhibition could restore the developmental competency of embryos impaired by sperm-derived oxidative stress. Consistent with this, the percentage of embryos that developed beyond the two-cell stage increased after WIP1 inhibition. However, these embryos were not able to develop to the blastocyst stage (Supplementary Table. S1). This is probably because DNA damage in sperm is high and exceed the maternal repair capability. Consistent with this, it has been reported that higher levels of sperm DNA damage are associated with failure to reach the blastocyst stage and embryonic loss between the embryonic genome activation and blastocyst stages (Simon et al., 2014; Garcia-Rodriguez et al., 2018). Given that double-strand breaks (DSBs) could be repaired by non-homologous end joining (NHEJ) that is considered error-prone by the absence of homologous templates (Hefferin and Tomkinson, 2005), it is also considerable that zygotic repair of paternal DSBs might not be sufficient to preserve genomic integrity to ensure normal embryonic development. Indeed, it has been reported that NHEJ is a predominant repair pathway in the zygotes (Fiorenza et al., 2001). Taken together, our results suggest that WIP1 inhibition efficiently reduces DNA damage derived from sperm in zygotes, improving developmental competence of embryos (Figure 4).

## Conclusion

Because DNA damage in germ cells induces adverse health effects in the progeny, the efficient DNA repair during gametogenesis and the early embryonic division after fertilization is essential to prevent transmission of DNA damage to the next generation. In particular, it is important to repair sperm DNA damage in the embryo after fertilization, because sperm have no DNA repair capability unlike oocytes. If not properly repaired, sperm DNA damage in zygotes can increase the risk of mis-rejoining, the generation of chromosomal rearrangements, and/or the formation of acentric fragments. Therefore, it is critical to repair sperm DNA damage before the first round of cell division in the zygote. In this study, we showed that WIP1 inhibition efficiently reduces sperm-derived DNA damage in zygotes by enhancing DNA repair capacity. Our data not only highlight the importance of maternal DNA repair during the early phases of mammalian development in assuring the genomic integrity of embryos, but also provide ways to improve maternal DNA repair capacity in zygotes. Considering that sperm DNA damage increases with age, our results have interesting implications for clinical settings, where culture medium could be supplemented with a WIP1 inhibitor to correct damaged DNA during assisted reproductive technology (ART) procedures.

## Data Availability

The original contributions presented in the study are included in the article/Supplementary Material, further inquiries can be directed to the corresponding author.
