# Supplementary material for: The Capacity to Repair Sperm DNA Damage in Zygotes is Enhanced by Inhibiting WIP1 Activity
Source: Front Cell Dev Biol. 2022 Apr 5;10:841327. doi: 10.3389/fcell.2022.841327 (PMC9037036; doi:10.3389/fcell.2022.841327)
Supplement: Supplementary file 1 [file DataSheet1.PDF]

Fig. S1

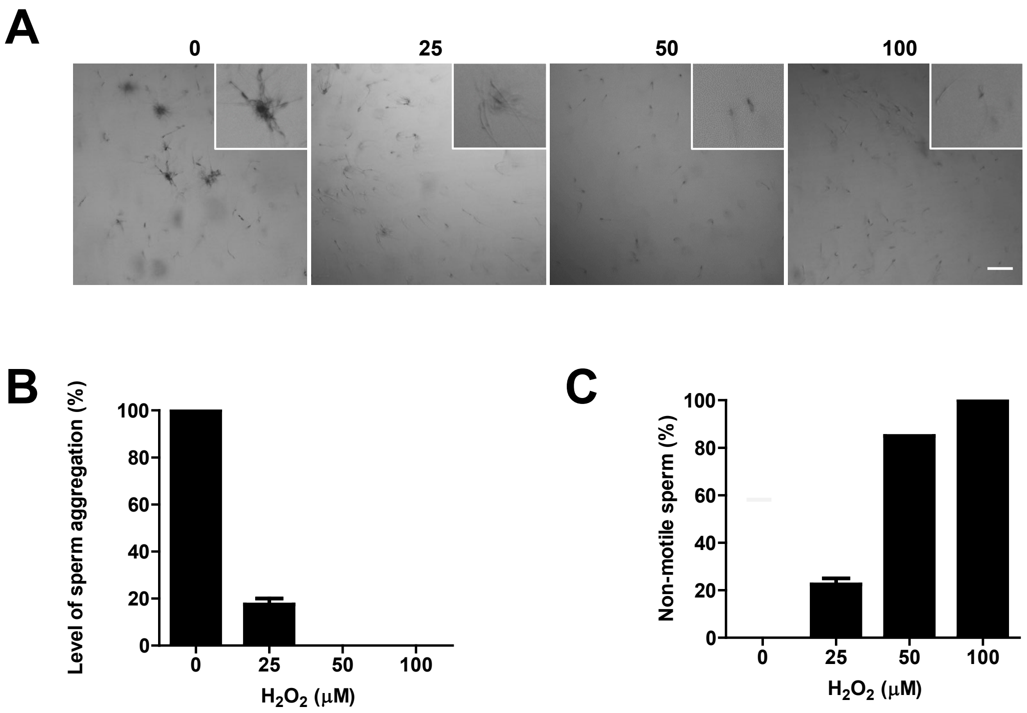

Fig. S1. Oxidative stress decreases sperm motility. (A) Representative images of sperm treated with 0, 25, 50 and 100 μM H_2O_2. Scale bar, 100 μm. (B) Levels of sperm aggregation. (C) Percentage of non-motile sperm.

Table S1. Rate of embryo development beyond 2-cell stage

Groups	Embryos*	2-cell (%)	4-cell (%)	Morula (%)	Blastocysts (%)
DMSO	76	12 (15.49 ± 2.77)[a]	5 (7.063 ± 3.70)[a]	0	0
GSK	75	25 (33.87 ± 0.84)[b]	14 (18.67 ± 5.91)[b]	4 (5.57± 5.55)	0

* At least three independent experiments were performed for each group.

[a,b] Values with different superscript symbols in the same column differ significantly ($P<0.05$)
